# Medical Ethics

**Published:** 1900-10-20

**Authors:** 


### MEDICAL ETHICS.

In the *International Journal of Ethics* for this month there is an article by Mr. Brudenell Carter on "Medical Ethics," which, taken on the whole, gives a fair and what we may call a gentlemanly view of the subject.

Bearing in mind the many years during which Mr. Carter had a seat on the General Medical Council, a special interest attaches to those portions of his article in which he deals with the limits within which the powers possessed by the Council are likely to be exercised in restraint of various practices which are regarded by many medical men as constituting "infamous conduct."

In endeavouring to define what sort of conduct may properly be considered so "infamous" in the words of the Medical Act as to justify expulsion from the Register, Mr. Carter says that this word certainly could not be applied to acts which are merely undesirable or even discreditable, but must be restricted to offences properly regarded as grave and serious.

At the present time there are many medical men who call aloud for the protection of the General Medical Council, and hold that that august body is acting negligently and is betraying the interests of the profession in not taking action against those who accept office in certain medical aid associations; and it is well known that various professional organisations which have endeavoured more or less successfully to enforce "what may be described as trades-union rules for the restraint of devices suggested by the stress of competition," such as "underselling," "touting," &c., are urgent in their desire that the Medical Council should declare such acts and devices "infamous," and should expel all who do such things. In regard to this Mr. Carter says that "it is idle to suppose that the English Legislature will ever consent to inflict disabilities upon men who choose to sell what skill or knowledge they possess at their own estimate of its value."

Put in such words, this proposition no doubt has an appearance of incontrovertibility, and so far as low fees are concerned we agree with Mr. Carter that the amount of a man's fee could never give ground for his expulsion from the register. But when a man has to live by his profession smallness of fee is necessarily correlated with largeness of practice, and one cannot doubt that a limit is sooner or later reached at which an element of fraud comes into the undertaking, and that the medical man who for the sake of making a living by very small fees undertakes an amount of contract work which cannot possibly be properly performed, he is guilty of something much more serious than the infraction of mere trades-union rules.

If it were the fact that the workman who paid his penny a week for contract medical attendance recognised and understood that he was not to expect full and proper medical services, however undesirable the arrangement might be, there would be no fraud. He would pay his penny, and would have to put up with his pennyworth. But we are not aware that there is any such suggestion. The phrase "medical attendance" has a certain meaning, and whatever else it includes it certainly involves an obligation upon the doctor to do his best. What this "best" means the patient has no knowledge. This is all left to the doctor's honour; and here lies the immorality of any contract which cannot be properly performed.

Against such contracts something more stringent than mere trades-union action may properly be invoked. Those, however, who ask for the interference of the powers in this regard must remember that the officers of medical aid associations are not the only sinners. If the complaints we hear from time to time as to the conditions of poor-law work in certain districts are true presentments of the facts, it may be doubted whether some who accept such work under such conditions might not equally be described as entering into an immoral contract in undertaking to do what they cannot possibly perform with full efficiency.

